# Giant cell tumor of the temporal bone – a case report

**DOI:** 10.1186/1472-6815-5-8

**Published:** 2005-09-15

**Authors:** S Balaji Pai, RM Lalitha, Kavitha Prasad, Saraswathi G Rao, K Harish

**Affiliations:** 1 Department of Neurosurgery, M.S. Ramaiah Medical College, Bangalore, India; 2 Department of Oromaxillofacial surgery, M.S. Ramaiah Medical College, Bangalore, India; 3 Department of Pathology, M.S. Ramaiah Medical College, Bangalore, India; 4 Department of Surgical Oncology, M.S. Ramaiah Medical College, Bangalore, India

## Abstract

**Background:**

Giant cell tumor is a benign but locally aggressive bone neoplasm which uncommonly involves the skull. The petrous portion of the temporal bone forms a rare location for this tumor.

**Case presentation:**

The authors report a case of a large giant cell tumor involving the petrous and squamous portions of the temporal bone in a 26 year old male patient. He presented with right side severe hearing loss and facial paresis. Radical excision of the tumor was achieved but facial palsy could not be avoided.

**Conclusion:**

Radical excision of skull base giant cell tumor may be hazardous but if achieved is the optimal treatment and may be curative.

## Background

Giant cell tumor (GCT) of bone is an uncommon primary bone neoplasm that usually occurs in the long bones. It is rarely encountered in the skull where it is preferentially seen to involve the sphenoid and the temporal bones. It is a benign neoplasm but can be locally aggressive. A tendency towards local recurrence and late malignant change with metastases especially to the lung has been reported [1,2]. Radical surgical removal is the preferred modality of treatment. We present a GCT of the temporal bone in a 26 year old male which was treated with radical surgery with a good outcome.

## Case presentation

### Clinical presentation

**Figure 1:**
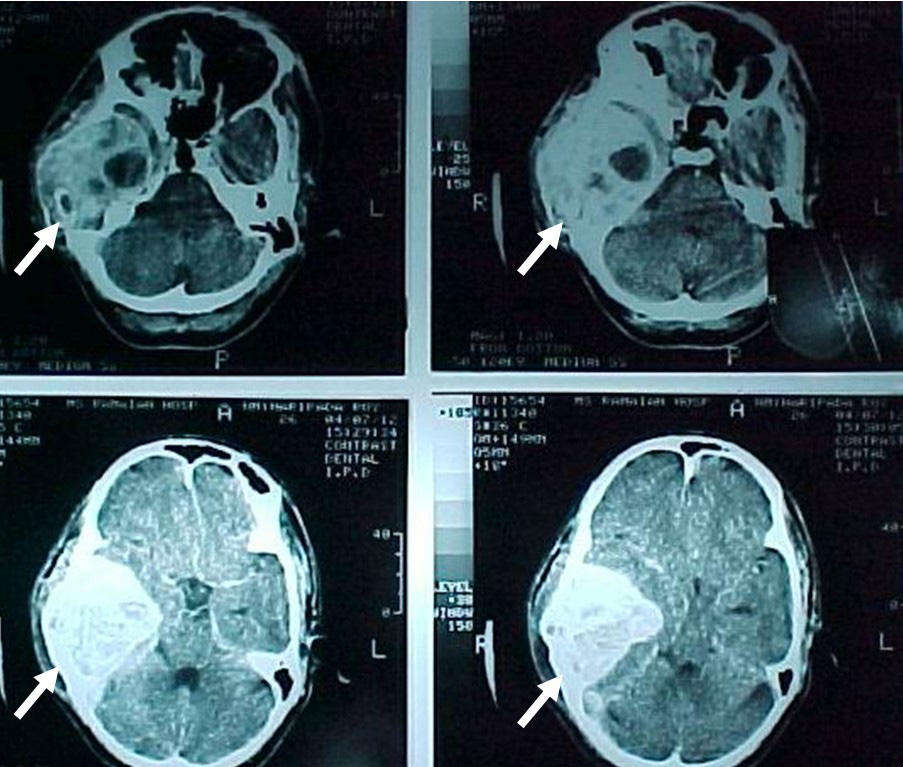
**Preoperative CT scan. **Preoperative axial CT scan which shows a large hyperdense tumor arising from the petrous portion of the right temporal bone with intracranial extension and impending uncal herniation.

**Figure 2:**
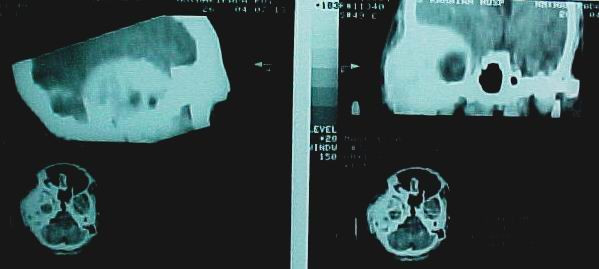
**Preoperative CT scan. **The figure shows the sagittal and coronal reconstruction of the tumor.

A 26 year old male was admitted with impaired hearing and tinnitus on the right side and swelling of the right temporal region which was gradually progressive for the last two years. His general physical examination was normal. Neurological examination revealed a severe right conductive hearing loss with a Grade II House-Brackman facial nerve paresis. A diffuse swelling was noted in the right temporal and preauricular region. CT scan of the brain showed a large well defined hyperdense contrast enhancing lesion originating from the right temporal bone – squamous and petrous portions with a large intracranial extension causing uncal herniation [Fig. 1 &2].

### Surgical management

**Figure 3:**
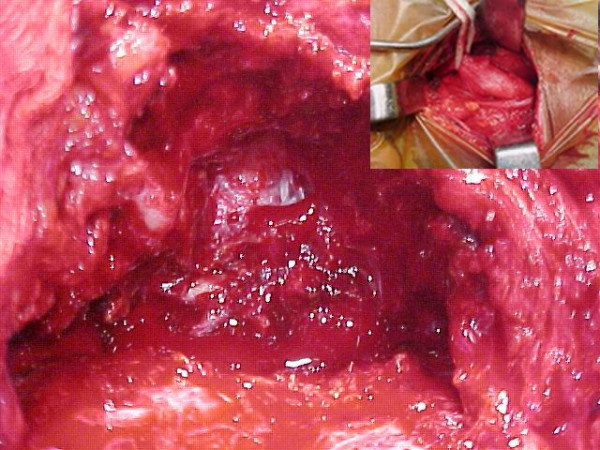
**Tumor exposure. **Right temporoparietal craniectomy and tumor exposure after right external carotid artery control (inset) and excision of the temporalis muscle. Tumor was friable, reddish brown and vascular.

**Figure 4:**
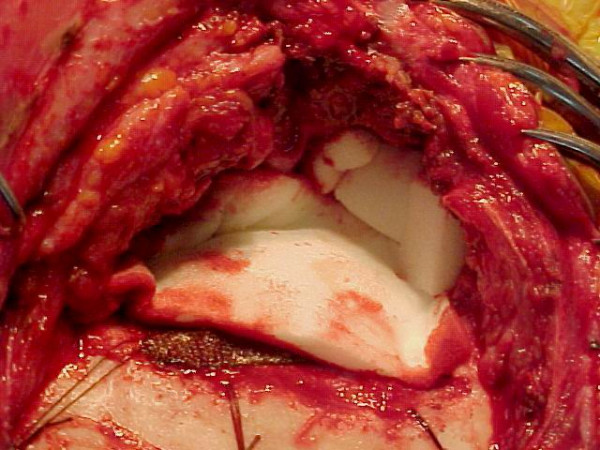
**After completion of tumor excision. **After radical excision of tumor (piecemeal) the defect is covered with gelfoam.

The patient was taken up for surgery with an intention of radical removal. Control of the right external carotid artery (ECA) was obtained in the neck [Fig. 3 inset]. Right frontotemporal scalp flap was raised. The temporalis muscle was seen to be infiltrated by the tumor and was excised seperately. The tumor was firm, reddish brown and vascular. It had destroyed the squamous temporal bone, lateral petrous portion, zygomatic arch and was seen invading the cranium pushing the temporal bone superiorly and medially along with the dura [Fig. 3]. Dura was not transgressed. Piecemeal total removal of the tumor was achieved with temporary clamping of the right ECA. The tumor was adherent to the dura but could be peeled off the dura [Fig. 4]. Biopsy was taken from surrounding bone, muscle and dura from 4 different sites. A drain was left in the large dead space created by the removal of the tumor. Cranioplasty was planned for a later date.

### Postoperative period and follow up

**Figure 5:**
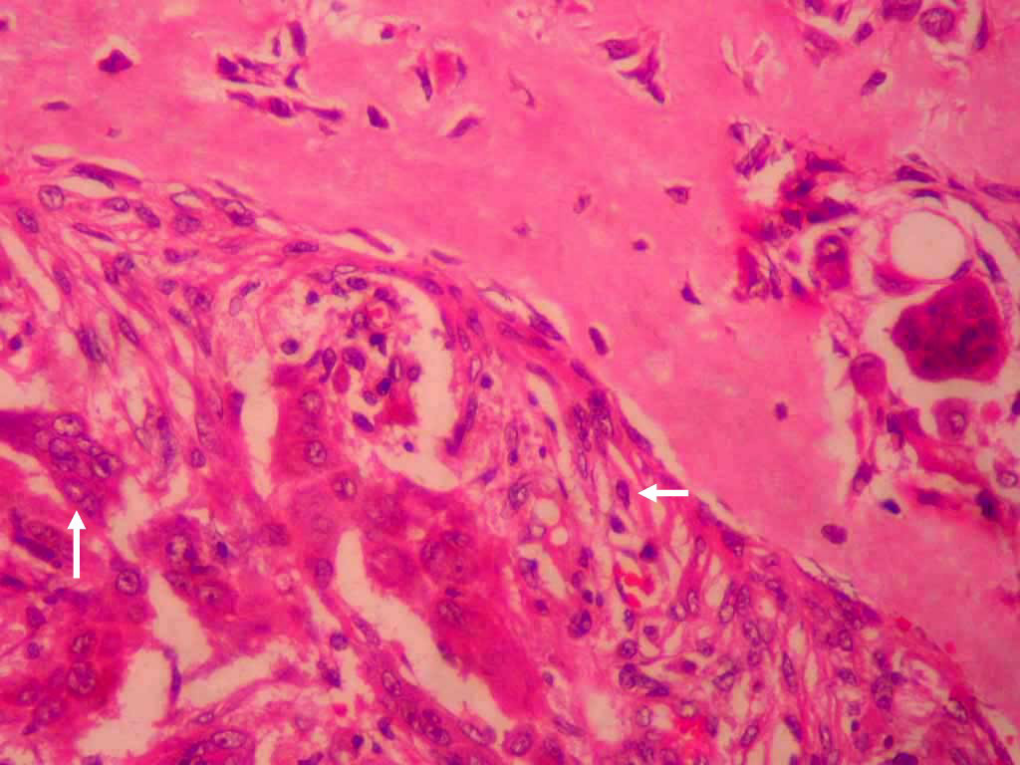
**Microphotograph of the tumor. **Microphotograph (250×) of the specimen in H & E stains showing neoplastic stromal cells and multinucleated giant cells. The stromal cells (horizontal arrow) are round to spindle shaped with moderate amount of eosinophilic cytoplasm and a single nucleus. They are mesenchymal in origin. The multinucleated giant cells (vertical arrow) are large in size with eosinophilic cytoplasm and a large number of nuclei.

**Figure 6:**
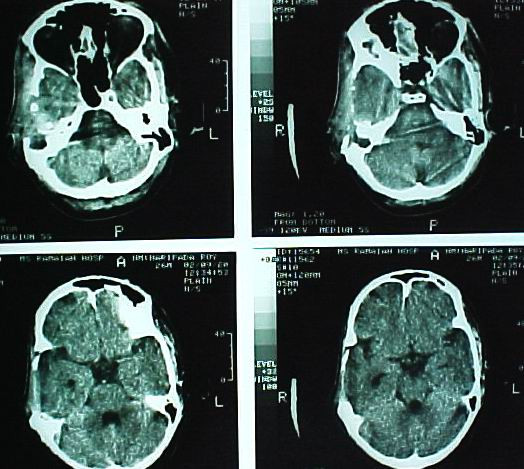
**Postoperative CT. **Postoperative CT scan of the patient showing total excision of the tumor and the normal alignment of the intracranial structures.

Postoperatively the patient developed a right total LMN VII nerve palsy (Grade VI House-Brackman). Hence a right tarsorrhaphy was done to prevent exposure keratitis one week after the first operation. At the same sitting the external auditory canal was also closed to prevent communication of the dead space in the cranium with the external auditory canal after confirming the absence of any collection in the intracranial dead space. The postoperative period was otherwise uneventful. Histopathological examination revealed a neoplasm composed of numerous osteoclast like giant cells amidst a background of mononuclear plump spindle cells suggestive of a GCT [Fig. 5]. The histopathological examination of the other 4 areas of bone, dura and muscle did not reveal any tumor infiltration. Postoperative CT scan confirmed a total excision of the tumor [Fig. 6]. Since a radical excision of the tumor had been achieved it was decided to defer radiotherapy. Three months after surgery patient was normal but for the deafness and facial palsy. Follow up CT at 6 months and 12 months did not reveal any recurrence.

### Discussion

Neoplasia of the skull bones are uncommon accounting for only 2.4% – 2.6% of all primary bone tumors [1]. The majority of giant cell tumors occur in the long bones usually the distal femur, proximal tibia and fibula, distal radius and ulna [3]. The skull is a rare location for GCT. In the cranium the sphenoid bone is the commonest site followed by the temporal bone [1,3-8]. This can be explained by the fact that the tumor genesis occurs in the endochondral bone instead of intramembranous bone [3,5-7]. The temporal bone has two main components – squamous and petromastoid. The squamous portion develops by intramembranous ossification, while the petreomastoid portion develops from cartilage (endochondral bone). GCTs are commonly seen to arise from the petromastoid portion as was noted in the present case. GCT is commonly seen in the 30–50 years age group with only 16% of patients below 20 years of age [6,9]. A mild female preponderance is seen but this is more pronounced in the younger age group [9]. Typically, the tumor presents as an enlarging mass associated with local pain over a period of few weeks to years [9]. GCT of the sphenoid may present with headache, visual field defects, blindness, diplopia, second through eighth cranial nerve dysfunction, endocrinopathy and change of mental status [4]. Temporal bone tumors present with pain usually behind the affected ear, deafness and facial weakness as in the present case [6]. Temporal bone GCT may invade the infratemporal fossa, paranasal sinuses and nasopharynx [6]. Intracranial extension as in our case may also be present. Dural penetration with invasion into the brain has also been seen [5,6]. Plain radiography shows radiolucent lesion of the skull and cannot be generally differentiated from other radiolucent lesions. On CT it is seen as a lytic lesion expanding the bony cortex [10]. Generally these tumors are contrast enhancing due to their vascular nature as seen in our patient.

These tumors generally tend to expand and attenuate the bony cortex rather than erode it [10]. GCT may be vascular and an external carotid angiogram may be required to demonstrate the arterial supply. In our patient the tumor was vascular but no angiogram was performed. However, temporary clamping of the external carotid artery during excision reduced the intraoperative bleeding. Grossly, these tumors are grey to yellow-brown, soft or firm and friable. Small cystic areas and grey-white necrotic foci may be seen. Microscopically, GCT consists of plump spindle shaped or ovoid cells with admixed multinucleated, cytologically benign giant cells. Variable numbers of benign multinucleated cells are seen amidst sheets of benign mononuclear spindle shaped cells with similar nuclear features. The nuclei are generally hypochromatic with inconspicuous nucleoli and mitotic figures are uncommon [4]. Histological differentiation of GCT may be challenging. The differential diagnoses consist of central giant cell granuloma (CGCG), aneurysmal bone cyst, chondroblastoma, hyperparathyroidism and fibrous dysplasia [8]. CGCG is a reactive bone lesion that occurs mainly in the jaws [11]. CGCG and GCT are histologically very similar and the main significant difference is the greater number of nuclei in the giant cells of the GCT [12,13]. CGCGs are distinguished from true GCTs by their fibrogenic, relatively acellular stroma, extensive osseous metaplasia and the clustering of giant cells around areas of hemorrhage or necrosis [5]. A key point in the differential diagnosis is that in GCT the stromal cells and giant cells resemble each other particularly with regard to their nuclei, whereas in giant cell reparative granuloma, the osteoclasts and the stromal cells of the fibroblastic type are distinctly different [14]. Jaffe has subclassified GCT into three grades but such a grading has not been found to correlate with subsequent tumor behaviour or sarcomatous transformation [1,5].

The precise ontogeny of GCT is unresolved. GCT and CGCG are histologically and pathogenetically similar [15]. Cell cycle associated proteins like MDM2, Ki-67 and PCNA have been seen to be widely expressed in CGCG and GCT. The percentage of Ki-67 and PCNA positive cells are higher in CGCG[11]. This means that CGCGs show a higher proliferative activity than GCTs. GCT cells are also seen to produce both MMP-9 and tumor necrosis factor-alpha (TNF-alpha) [16]. Studies suggest that TNF-alpha secreted by the multinucleated giant cells up-regulates MMP-9 expression in GCT stromal cells by the induction of certain transcription factors, which in turn enhanced the rate of transcription of MMP-9 gene. An essential cell-cell interaction in the regulation of MMP-9 expression exists in GCT [16]. Although it is the giant cell which is the most prominent feature of these lesions it is the mononuclear spindle cell which is the proliferating cell. Several pathways to induce osteoclast like giant cell formation from monocytes have been reported. The spindle cell recruits monocytes and induces them to differentiate into osteoclastic giant cells through release of cytokines [15,17]. Receptor activator of nuclear factor kappa B (RANK) ligand is also reported to play a crucial role in osteoclastic cell genesis [17-19]. It is possible that the soluble RANKL is released from the tumor derived cells and the soluble factor interacts with RANK expressed in monocytes resulting in osteoclast-like cell formation in cooperation with macrophage colony stimulating factor secreted from the cells [17]. SDF-1 has also been incriminated as one of the significant chemo-attractant factors involved in the recruitment of hematopoietic osteoclast precursor cells during tumor-induced osteoclastogenesis [20]. The histopathogenesis of GCT and CGCG thus appears to be identical but the biological and clinical behavior of GCT is more aggressive than the latter.

The treatment of choice is complete surgical excision which if achieved can be curative [1,5,9]. However the skull base location of these tumors can make total surgical excision hazardous and not possible [5,6]. The role of adjuvant radiotherapy in eliminating residual tumor tissue is controversial. Some authors claim that GCT is not radiosensitive and radiation may provoke a sarcomatous transformation in the residual tumor tissue [3,5]. However, other authors recommend a single course of moderate dose super voltage radiation in achieving a high success rate and at the same time lowering the likelihood of malignant transformation [4-6,8]. Radiotherapy remains the only option for unresectable tumors. In our patient radiotherapy was deferred for the present as radical surgical excision was achieved with no residual tumor.

Treatment of GCT and CGCG with calcitonin and osteoprotegrin has received attention. Osteoclasts express calcitonin receptors and can be inhibited by calcitonin. In GCT and CGCG, tumor giant cells and their precursors also express calcitonin receptors. Clinical studies on treatment of CGCG with calcitonin have shown positive results probably due to control of osteoclastogenesis [15]. Osteoprotegrin ligand was also selectively overexpressed in GCTs and may indicate another possible target to which antitumor therapy could be directed [21]. Osteoprotegrin influences osteoclastogenesis and may be used for the treatment of GCT and CGCG. Osteoprotegrin is a "decoy" receptor for RANKL and therefore inhibits the RANK-RANKL interaction, which is a necessary step in the osteoclastogenesis. This is the rationale for a probable therapeutic use of Osteoprotegrin. Its utility is however unproven [15].

GCT can recur especially where only a curettage is employed [6,7]. Prosser et al recommend primary curettage for intraosseous giant-cell tumors without adjuvant treatment or filling agents, but tumors with soft tissue extension or with local recurrence require more aggressive treatment [22]. Metastases occur in only 2% of cases and are usually to the lungs but spread to rare areas like lymph nodes, mediastinum, skin, scalp and pelvis has been reported [2,9]. Our patient is on regular clinical follow up every 3 months and 6 monthly CT scan. He is recurrence free at the end of 12 months.

## Conclusion

Giant cell tumor involving the petrous portion of the temporal bone is rare. A radical excision if possible is the optimal treatment. Adjuvant radiotherapy is controversial and should be reserved for residual tumor and unresectable tumors. Newer modalities like calcitonin and osteoprotegrin are being investigated.

## Competing interests

The author(s) declare that they have no competing interests.

## Authors' contributions

**SBP **performed the craniectomy and excised the tumor and drafted the manuscript.

**RML **and **KP **assisted in the surgery.

**SGR **did the histopathological examination and helped in drafting the manuscript.

**KH **helped in drafting the manuscript and references

All authors have read and approved of the manuscript.

## Consent to publish

Patient's consent was obtained to publish the manuscript.

## Pre-publication history

The pre-publication history for this paper can be accessed here:
